# What’s new in the ESC 2018 guidelines for arterial hypertension: The ten most important messages

**DOI:** 10.1007/s00508-018-1435-8

**Published:** 2019-02-04

**Authors:** Jutta Bergler-Klein

**Affiliations:** Department of Cardiology, Medical University of Vienna, Waehringer Guertel 19–20, 1090 Vienna, Austria

**Keywords:** Systolic blood pressure, Antihypertensive treatment, diastolic blood pressure, hypertension in coronary disease, hypertension and heart failure

## Abstract

The new guidelines on hypertension of the European Society of Cardiology (ESC) 2018 have refined the treatment cut-offs and therapy decisions in adults. This review highlights important recommendations of the guidelines and also on the situation of hypertension in Austria. The general treatment targets of blood pressure have been lowered to at least 130/80 mmHg for most patients. The definition of hypertension is specified as a repeated systolic blood pressure in the office of ≥140 and or diastolic BP ≥90 mmHg. For home blood pressure monitoring, an average value of ≥135/85 mmHg is now defined as hypertension. Ambulatory 24h-blood pressure measurement is recommended for diagnosis of hypertension and to demask lack of nocturnal blood pressure dipping. Whether drug treatment should be initiated immediately or after a delay with lifestyle intervention is focused on the individual high or low cardiovascular risk of the patients and the degree of hypertension. For most patients a combination therapy with single pill is now recommended as initial therapy for hypertension from the start. The salt consumption should be reduced in the majority of patients. The new guidelines have clarified the treatment of hypertension in different comorbidities.

## Introduction

The new 2018 guidelines on hypertension of the European Society of Cardiology (ESC) have refined the treatment cut-offs and therapy decision-making in adults [1]. This review focuses on the most important messages and also on the situation in Austria.

## Ten most important messages

### 1. What is defined as hypertension?

The definition of hypertension is now specified as a constant, repeated systolic blood pressure (SBP) in the office of ≥140 mm Hg and or diastolic BP (DBP) ≥90 mm Hg. Before office determination patients should be seated quietly for 5 min.

A 24 h ambulatory BP (ABPM) is strongly encouraged in all patients for screening and diagnosis of hypertension. It is important to note that in ABPM a lower value with an average of ≥130/80 mm Hg is already defined as hypertension. For home BP monitoring, an average value of ≥135/85 mm Hg is now defined. These values enable patients and physicians to choose from the available diagnostic tools; however, the different cut-offs for the definitions should be considered. It also empowers patients and their own responsibility by home measurements to detect and monitor hypertension.

### 2. Target range for blood pressure treatment

The general treatment targets of BP have been lowered to at least 130/80 mm Hg for almost all patients. This is in line with the recommendations of the American ACC/AHA guidelines for hypertension [2]. In all patients that can tolerate treatment, the office SBP should be lowered to <140 mm Hg. Office diastolic BP should in general be lowered to <80 mm Hg. In patients younger than 65 years old, office systolic BP lower than 130 mm Hg should be aimed for, but not below 120 mm Hg. In older patients over 65 years, and in old patients up to age 80 years who are capable of an independent life style and are not frail, a target SBP of 130 mm Hg but not below 130 mm Hg is recommended. In old patients over >80 years, treatment should generally be initiated in an office SBP ≥160 mm Hg. In frail patients individual decisions with gentle reductions are advised according to the benefit expectations of treatment. Importantly, the lower thresholds for BP treatment are now also clearly defined. Systolic BP should not be lowered to below 120 mm Hg. Diastolic BP should not be lowered to below 70 mm Hg. Therefore, clear target ranges have now been defined with lower BP cut-offs where antihypertensive treatment should not go beyond these values. When starting antihypertensive drugs, the first objective should be to lower BP to <140/90 mm Hg in all patients. If the treatment is then well-tolerated, BP should be targeted to 130/80 mm Hg or lower in most patients; however, treated SBP should not be targeted to <120 mm Hg as stated above and DBP not below 70 mm Hg.

### 3. Grading of degree of hypertension

**Fig. 1:**
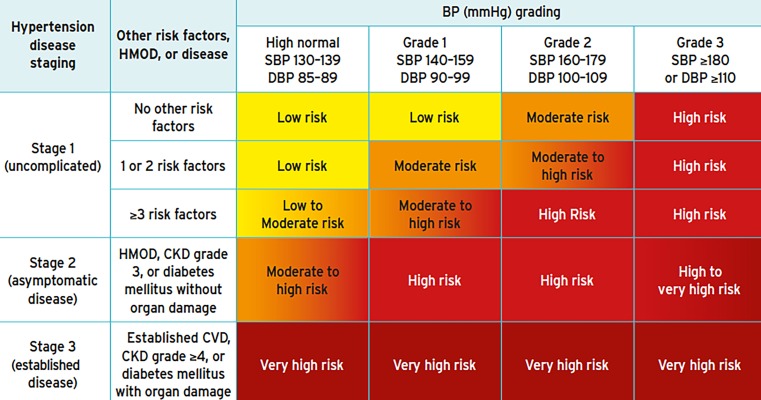
Staging of hypertension according to blood pressure and cardiovascular risk by the SCORE system [1]. *CKD* chronic kidney disease, *CV* cardiovascular, *DBP* diastolic blood pressure, *HMOD* hypertension‑mediated organ damage, *SBP* systolic blood pressure, *SCORE* Systematic COronary Risk Evaluation. Source and © [3]. Reproduced by permission of Oxford University Press on behalf of the European Society of Cardiology. www.escardio.org/Guidelines/Clinical-Practice-Guidelines/Arterial-Hypertension-Management-of. This figure is not included under the Creative Commons CC BY license of this publication

The degree of hypertension (grades 1–3) determines the initiation of treatment and the individual cardiovascular risk of the patient. Fig. 1 depicts the grades according to BP levels.

### 4. Treatment initiation: cut-offs revisited in high or low risk

**Fig. 2:**
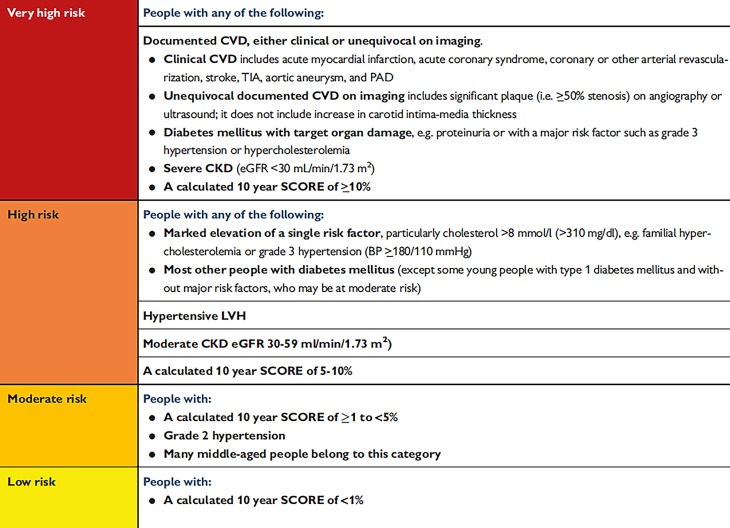
The 10-year cardiovascular risk categories by the European Systematic COronary Risk Evaluation system (SCORE) [1]. Source and © [3]. Reproduced by permission of Oxford University Press on behalf of the European Society of Cardiology. www.escardio.org/Guidelines/Clinical-Practice-Guidelines/Arterial-Hypertension-Management-of. This figure is not included under the Creative Commons CC BY license of this publication

Whether pharmaceutical treatment should be initiated immediately or after a delay with life style interventions is focused on high or low cardiovascular risk of the patients (Fig. 2).

In lower risk patients with grade 1 hypertension (defined as office BP 140–159/90–99 mmHg, see Fig. 1) and without end organ damage aged up to 80 years, treatment should be started after a trial of life style changes e. g. for 3–6 months. On the other hand, for high risk patients with grade 1 hypertension (140–159/90–99 mmHg) medical drug therapy should be initiated immediately without delay. Patients with grade 2 (160–179/100–109 mm Hg) or grade 3 hypertension (≥180/≥110 mm Hg) should receive immediate antihypertensive drug treatment along with life style intervention. Life style changes are enforced in the current guidelines, whether before begin as well as always during ongoing medical treatment. They include smoking cessation, weight loss, sodium restriction, moderation of alcohol, exercising, and healthy food with high amounts of vegetables and fruits.

### 5. Sodium restriction, alcohol

A maximum sodium intake of 2.0 g per day (about 5.0 g salt, one small teaspoon) in the general population and in all hypertensive patients is now recommended. Adding salt and processed foods with hidden salt should be avoided, as they involve 80% of salt consumption. The BP lowering effect of sodium restriction is endorsed as greater in black patients and in older patients and concomitant diabetes or chronic kidney disease. Importantly, sodium restriction may reduce the necessary number or dose of antihypertensive drugs. For cardiovascular event reduction, a controversial J‑shaped curve for sodium intake has been suggested in meta-analyses [4]. Overall, lowering the sodium intake is targeted at patients with manifested hypertension. In hypertensive men, alcoholic drinks should be limited to 14 units per week, in women to 8 units per week (1 unit corresponds to 1/8 l of wine or 1/4 l of beer). Alcohol-free days during the week and avoidance of binge drinking are advised.

### 6. Two in one approach: single pill dual drug from the start

The new guidelines emphasize that medical treatment should in general be started straight away with a combination pill of two drugs as usual care. In most patients the currently recommended lower BP targets will not be reached without modern dual therapy. Furthermore, a single pill approach with optimal retardation drug formulation for a long plasma half-life will increase the medical adherence of the patients. If BP targets are not reached, augmenting to a single pill with 3 drugs is preferred.

### 7. Simplified drug algorithm

For most patients, a combination of a renin-angiotensin system (RAS) blocker, either an angiotensin-converting enzyme inhibitor (ACEI) or angiotensin receptor blocker (ARB), with a calcium channel blocker (CCB) or thiazide/thiazide-like diuretic (TH) such as chlorthalidone and indapamide is preferred as initial therapy. If three drugs are required to lower BP to targets, a combination of an ACEI or ARB with a CCB and a TH-diuretic are the right choice, again in a single pill combination. Beta-blockers are only recommended in specific indications such as angina, after myocardial infarction, heart failure with reduced ejection fraction or heart rate control in arrhythmias. Beta-blockers should be combined with any of the other major antihypertensive drug classes (RAS blockers, CCB, diuretics). A combination of two RAS blockers (ACEI and ARB) is not recommended. In resistant hypertension, especially the addition of spironolactone (25–50 mg o.d.) is recommended. Also, another diuretic, an alpha-blocker or beta-blocker can be added. Hypertension is defined as resistant when the recommended treatment fails to lower office SBP and DBP to <140 mm Hg and/or <90 mm Hg, respectively and is confirmed by 24 h ABPM or home BP measurements despite confirmed drug adherence. Optimal doses of tolerated drugs and three or more drugs are recommended along with life style changes. Secondary causes of hypertension should be excluded. BP resistance can be mimicked by severe brachial artery calcification, white coat hypertension, wrong measurements, e. g. with too small cuffs, and of course a lack of patient therapy compliance.

### 8. Special considerations in special groups

Treatment thresholds of office BP are defined as ≥140/≥90 mm Hg and are the same in hypertensive patients with additional diabetes, coronary artery disease (CAD), chronic kidney disease (CKD), stroke or transient ischemic attack (TIA); however, in very high-risk patients with CAD, previous stroke or TIA, treatment may be considered already in high–normal SBP of 130–<140 mm Hg. In patients older than 80 years, a threshold of ≥160/≥90 mm Hg is advised for all groups, equally in diabetes, CAD, CKD or stroke.

#### Coronary disease

In CAD, diastolic BP should not be lowered <70 mm Hg as myocardial perfusion may be impaired in lower values [5]. In CAD, treatment is already recommended at the threshold of high-normal BP of 130–139/85–89 mm Hg, as these patients are considered to be at very high risk.

#### Diabetes

For patients with diabetes, the same treatment targets are recommended for an office SBP target of 130 mm Hg or lower. SBP should not be lowered to <120 mm Hg. DBP target should be <80 mm Hg. In older patients ≥65 years the SBP target range is 130–140 mm Hg if tolerated. A variable visit to visit BP should be noted due to associated increased cardiovascular and renal risk. Caution is emphasized in autonomic polyneuropathy concerning postural or orthostatic hypotension. Nocturnal BP should be assessed by 24 h ABPM or in order to detect hypertension in apparently normotensive diabetic patients.

#### Chronic kidney disease

The RAS blockers (ACEI or ARB) are endorsed as more effective in reducing albuminuria than other antihypertensive drugs. The guidelines recommend a RAS blocker and CCB as the initial regimen drugs. In both diabetic or non-diabetic CKD, the SBP target is 130–139 mm Hg. Individualized treatment is advocated according to electrolytes. The use of loop diuretics is recommended when the estimated glomerular filtration rate (eGFR) is <30 ml/min/1.72 m^2^, as thiazide/thiazide-like diuretics are less effective or ineffective at this level. There is risk of hyperkalemia with spironolactone, especially when eGFR is <45 ml/min/1.72 m^2^ or baseline K^+^ ≥4.5 mmol/l.

#### Heart failure

In hypertensive patients with preserved or reduced ejection fraction (EF), antihypertensive treatment should be considered if BP ≥140/≥90 mm Hg. If antihypertensive treatment is not needed, the treatment of heart failure (HF) should follow the current ESC HF guidelines [6]. In HF with reduced EF the initial antihypertensive regimen advocates an ACEI or ARB (or angiotensin receptor/neprilysin inhibitor as indicated by guidelines) plus a TH-diuretic (or loop diuretic in edema), plus a beta-blocker. The second step adds the mineralocorticoid receptor antagonists spironolactone or eplerenone. It is emphasized not to use non-dihydropyridine CCBs, such as verapamil or diltiazem. Although in general, actively lowering the BP below 120/70 mm Hg should be avoided, patients may achieve lower values due to HF guideline-directed medications, which if tolerated should be continued.

#### Pregnancy

For pregnant women the special considerations are outlined in the new pregnancy guidelines in cardiovascular disease [7]. It is important to follow the compelling contraindications of specific antihypertensive drugs, especially ACEI and ARBs in pregnancy. Beta-blockers may be considered alternatively in women planning pregnancy or already pregnant, although fetal and neonatal bradycardia have been described. Hypertension is defined as office values of SBP ≥140 mm Hg and/or DBP ≥90 mm Hg. The classification of hypertension in pregnancy is mild if BP is 140–159/90–109 mm Hg, and severe if ≥160/110 mm Hg [1, 7]. The different entities include pre-existing hypertension, gestational hypertension, pre-existing plus superimposed gestational hypertension with proteinuria, pre-eclampsia and antenatally unclassifiable hypertension. All pregnant women should be screened for proteinuria early to detect renal disease and in the second half of pregnancy for diagnosis of pre-eclampsia.

### 9. What else for risk reduction?

Statins should in general be prescribed in hypertensive patients with established coronary disease or in moderate to high cardiovascular risk by SCORE evaluation (Fig. 2) but are also recommended already in low to moderate risk. Low dose aspirin is not recommended for primary prevention in patients without cardiovascular disease. Antiplatelet therapy is indicated in hypertensive patients for secondary prevention e. g. after myocardial infarction or stent intervention.

### 10. Renal denervation not recommended

The use of device-based interventions such as carotid baroreceptor stimulation with pulse generator or baroreflex amplification stent device implantation, as well as catheter-based renal denervation for reduction of sympathetic tone is not recommended for the routine treatment of hypertension. Currently, not enough evidence for efficacy and safety is considered to be available.

## Discussion

The new ESC guidelines have lowered the treatment target to a BP of 130/80 mm Hg. The definition of hypertension is set at systolic BP ≥140 mm Hg and/or diastolic BP ≥90 mm Hg. This has caused some discussion, although the guidelines clearly aim at especially lowering the high-risk profiles of patients with concomitant cardiovascular diseases, e. g. coronary disease or diabetes [8].

Importantly, the new guidelines have also introduced lower BP thresholds, below which treatment should not be continued, in general SBP 120 mm Hg as lower systolic threshold. Therefore, the current ESC guidelines have defined clear BP target ranges: SBP of 120–130 mm Hg in patients younger than 65 years old and 130–139 mm Hg in those older than 65 years and even over age 80 years if tolerated. Diastolic BP should not be lowered below 70 mm Hg. Therefore, the diastolic target range is now 70–79 mm Hg in all patients. It has been realized that excessive BP lowering causes more adverse events and higher discontinuation rates by patients [9]. A dilemma in hypertension treatment remains in discrepancy of systolic or diastolic hypertensive BP values, as both components cannot be regulated independently in some patients [8].

A single pill prescription with two or more drug ingredients is now confirmed as usual care when initiating antihypertensive treatment right from the start. This regimen will increase patient compliance and reduce side effects, as a relatively lower dosage of individual drugs may be applied with better galenics. Beta-blockers are now only recommended in special situations, e. g. after myocardial infarction, reduced ejection fraction heart failure and arrhythmias.

### Austrian perspective

A wider use of out of office measurements is now recommended. Ambulatory 24 h BP measurement is useful to demask nocturnal hypertension and lack of adequate dipping. In Austria, ABPM is not reimbursed by all public healthcare systems so far and will need to be established further. The salt consumption should be reduced in the majority of patients. The usual consumption of sodium is 3.5–5.5 g per day (9–12 g of salt), depending on country or region. In Austria, half of the adults consume more than 2 teaspoons of salt per day [10]. There is a causal relationship between the pressor effect of excessive sodium intake >5 g per day and an increased prevalence of hypertension and SBP rise with age [11]. In Austria as in other European countries the food industry must be involved in the future in the attempt to decrease hidden sodium consumption.

High altitudes above 3000 m and possibly 2000 m may contribute to aggravation of hypertension, which must be considered especially in the alpine regions of Austria [1, 12]. Frequent BP measurements and intensified antihypertensive medication adaptation are recommended, e. g. during holidays in mountain areas.

## Conclusion

The new ESC guidelines have clearly defined therapeutic targets with lower thresholds. In most patients a BP goal of at least 130/80 mm Hg is recommended, but not below 120/70 mm Hg. Life style interventions are enforced in all stages of hypertension.
